# Breeding in the pandemic: short-term lockdown restrictions in a European capital city did not alter the life-history traits of two urban adapters

**DOI:** 10.1007/s11252-022-01309-5

**Published:** 2022-12-14

**Authors:** Michela Corsini, Zuzanna Jagiello, Michał Walesiak, Michał Redlisiak, Ignacy Stadnicki, Ewa Mierzejewska, Marta Szulkin

**Affiliations:** 1 Centre of New Technologies, University of Warsaw, ul. Banacha 2c, 02-097 Warsaw, Poland; 2 Department of Zoology, Poznań University of Life Sciences, Wojska Polskiego 71C, Poznań, 60-625 Poland; 3 Mammal Research Institute, Polish Academy of Sciences, ul. Stoczek 1, 17-230 Białowieża, Poland; 4 Faculty of Biology, University of Gdansk, Bird Migration Research Station, ul. Wita Stwosza 59, 80-308 Gdansk, Poland; 5 Artes Liberales, University of Warsaw, ul. Nowy Świat 69, 00-046 Warsaw, Poland

**Keywords:** Lockdown, SARS-CoV-2, Urbanisation, Human presence, Avian life-history traits, Tree cover

## Abstract

Humans are transforming natural habitats into managed urban green areas and impervious surfaces at an unprecedented pace. Yet the effects of human presence *per se* on animal life-history traits are rarely tested. This is particularly true in cities, where human presence is often indissociable from urbanisation itself. The onset of the SARS-CoV-2 outbreak, along with the resulting lockdown restrictions, offered a unique, “natural experiment” to investigate wildlife responses to a sudden reduction in human activity. We analysed four years of avian breeding data collected in a European capital city to test whether lockdown measures altered nestbox occupancy and life-history traits in terms of egg laying date, incubation duration and clutch size in two urban adapters: great tits (*Parus major*) and blue tits (*Cyanistes caeruleus*). Lockdown measures, which modulated human presence, did not influence any of the life-history traits investigated. In contrast, the interaction between year and tree cover, a distinct ecological attribute of the urban space, was positively associated with clutch size, a key avian life-history and reproductive trait. This highlights the importance of inter-year variation and habitat quality over human activity on urban wildlife reproduction. We discuss our results in the light of other urban wildlife studies carried out during the pandemic, inviting the scientific community to carefully interpret all lockdown—associated shifts in biological traits.

**Supplementary Information:**

The online version contains supplementary material available at 10.1007/s11252-022-01309-5.

## Introduction

Humans are ecosystem engineers, capable of rapidly transforming natural habitats into managed green areas and impervious surfaces such as buildings, infrastructural networks and other built-up structures (Smith 2007). As such, humans affect directly and indirectly all living biota across a wide spectrum of habitats on Earth, and are thus identified as a “hyper-keystone species” (Worm and Paine 2016).

Ever since the Industrial Revolution, and especially over the past few decades, human ecosystems were characterised by an exponential growth of cities and towns worldwide, coupled with a steady migration of people from semi-natural, rural and marginal areas, to more urbanised settlements (Grimm et al 2008; Seto et al. 2011). Along with the urbanisation process, the footprint of human activities is now influencing all dimensions of the natural world, and is an undeniable threat to biodiversity (Grimm et al. 2008; McDonnell and Hahs 2015; Fenoglio et al. 2021; Sol et al. 2014). As such, cities are a valuable case study of ecological and evolutionary change as they rapidly induce novel selective pressures on animal and plant species (Szulkin et al. 2020a). These species may decline (urban avoiders), spread (urban exploiters) or thrive / survive (urban adapters) within these novel environments (Palacio 2020; Fischer et al. 2015; McKinney 2006). Urban adapters, such as great tits (*Parus major*) and blue tits (*Cyanistes caeruleus*), became valuable case studies to determine whether urban populations differ from their rural counterparts in terms of genotype, physiology or behaviour, and to identify what main components of the urban landscape cause such variation (Chamberlain et al. 2009). Earlier studies conducted on these two species reported differences in terms of life-history traits and reproductive success between urban and rural populations (Chamberlain et al. 2009), often identifying chemical (Isaksson 2010; Chatelain et al. 2021), light (Spoelstra et al. 2018; Ouyang et al. 2017; Kempenaers et al. 2010) and sound pollution (Dominoni et al. 2020; Mockford and Marshall 2009) as main drivers of these differences. Other studies emphasized the negative and pervasive effect of built up areas, infrastructural networks and, more generally, impervious surfaces on avian fitness (Corsini et al. 2017, 2020; Satgé 2019). Yet, very few studies tested whether human presence *per se* was linked to changes in avian trait distribution in urbanised contexts, and the results obtained, particularly in tit species, are not homogenous (Corsini et al. 2017; Remacha et al. 2016; Hutfluss and Dingemanse 2019). For example, a study conducted in a European capital city (Warsaw) did not find any association between human presence, measured at the nestbox level, and any of the life-history traits investigated in great and blue tits (Corsini et al. 2017). In contrast, blue tit nestlings reared in a Spanish natural forest developed slower when hatching occurred on weekends - thus, when more visitors where present at the study site - or when they hatched closer to recreational facilities, ultimately fledging with poorer body condition (Remacha et al. 2016). Similarly, in a urban population of great tits in Germany (12 study plots between Starnberg and Herrshing), birds were more likely to breed further away from highly frequented paths and those that bred closer were found to lay smaller clutches (Hutfluss and Dingemanse 2019).

Certainly, a considerable limitation in studying the effects of human presence *per se* on these two species or, more generally, on wildlife across the urban mosaic, stems from the unfeasibility to disassociate human presence from urbanisation in general, which is a wider and more complex ecological process. Consequently, it was impossible to date to exclude the constant presence of humans from urban areas – where “crowds” are the norm, and where fine-scale heterogeneity of human presence in the urban mosaic is also known to be repeatable over time and space (Corsini et al. 2019, but see Galván et al. 2014; Møller and Mousseau 2011; Mousseau and Møller 2012; Seress et al. 2021). As the presence of humans in urban green areas overlaps with the breeding season of great tits and blue tits across the urban mosaic, knowledge on how these two urban adapters respond in terms of life-history strategy to a sudden disappearance of the “human component” remains little explored (but see Seress et al.; 2021). While answering such a research question could be perceived as utopian especially in cities, 2020 proved us wrong.

Along with the spread of the novel coronavirus disease, the World Health Organization (WHO) declared SARS-CoV-2 a  zoonotic pandemic on the 11th of March 2020 (WHO; 2020). This kick-started a cascade of governmental actions worldwide aimed at containing virus transmission (Anderson et al. 2020). Most of these were realised through the cancellation of public events and the immediate interruption of any type of gatherings characterised by high human densities, be it commercial or social events. Although the timing and strength of lockdown restrictions imposed by each government differed between countries, quarantine and stay-at-home orders considerably reduced the use of public transports and the flow of people within and outside of cities during the first pandemic wave of infections, creating newly emptied soundscapes even where crowds and chaos were routine (Derryberry et al. 2020). This new realm, defined as the “*Anthropause*”, offered a unique opportunity for scientists to investigate wildlife responses to lockdown measures in cities where, while for the first time the outdoors were emptied from their hyper key-stone species (Rutz et al. 2020) (Table S1).

Shortly after the SARS-CoV-2 outbreak, media outlets reported unusual sightings of animal species that were rarely (if ever) observed in cities; similarly social media were flooded with photos of wildlife in the urban space. The scientific community immediately started to investigate (to the best of lockdown regulations for any specific region) the consequences of lockdown restrictions on wildlife biology by collecting data in the field or through the direct observations of citizens (i.e., citizen-science projects and online platforms, Table S1). As an example, the majority of the earliest studies here reported (9 out of 14, Table S1) conducted on the effect of the SARS-CoV-2 lockdown focused on behavioural patterns of animal communities, most often quantified in terms of sightings (Vardi et al. 2021; Manenti et al. 2020; Quesada-Rodríguez et al. 2021). Analyses were generally performed by comparing pre-lockdown and lockdown periods for the recorded observations, and emphasized an increased trend for uncommon “species occurrence” in areas where lockdown restrictions were implemented (e.g. (Vardi et al. 2021; Manenti et al. 2020; Soh et al. 2021)). However, responses were not uniform across all species: in some cases, no difference pre—and during lockdown was noted (Vardi et al. 2021; Manenti et al. 2020; Soh et al. 2021), while in other cases, the directionality of the association was opposite to that expected (e.g. fewer sightings were reported during than before lockdown) (Vardi et al. 2021; Soh et al. 2021). For instance, some urban exploiters during lockdown decreased in number within certain urban areas: such changes may be related to the “absence” of anthropogenic food resources caused by this novel circumstance (Soh et al. 2021).

As the majority of studies reporting the impact of human lockdown on wildlife during the SARS-CoV-2 pandemic relate to behavioural traits, data on the impact of the pandemic on animal life-history and / or reproductive traits remains scarce (Manenti et al. 2020; Quesada-Rodríguez et al. 2021; LeTourneux et al. 2021). In fact, despite the presumed beneficial effects of lockdown on urban wildlife, only two studies (out of the 13 here described, Table S1) to date report a positive association between reproductive traits and implemented lockdown measures (e.g., increased hatching success in Leatherback sea turtles *Dermochelys coriacea* (Quesada-Rodríguez et al. 2021), and increased clutch size in common swifts *Apus apus* (Manenti et al. 2020)). In addition, there is only one study testing the effect of lockdown measures on great tit reproduction, which reported no association between the *Anthropause* and avian life-history traits (see (Seress et al. 2021)). Therefore, implications of the *Anthropause* on wildlife life-history variation in urban populations remain largely unexplored. To address this knowledge gap, we tested whether lockdown restrictions introduced during the SARS-CoV-2 pandemic in a European capital city (Warsaw, Poland) were associated with changes in nestbox occupancy patterns and life-history trait variation in two urban adapters: great tits *Parus major* and blue tits *Cyanistes caeruleus*.

The life-history traits investigated here included nestbox occupancy, lay date, clutch size and incubation duration. We focused on these traits as these were expressed during —or shortly after the period of the strictest lockdown measures in Poland, which lasted from the 1^st^ until the 20^th^ of April, 2020 (Dziennik Ustaw Rzeczypospolitej, 2019, Legislation nr 566 and nr 697). Within this time window, people were not allowed to access green areas or more generally - recreational areas, including protected areas and natural reserves. While most of the public facilities were closed and people switched to remote working, access to grocery stores and pharmacies and essential family care were the only allowed activities that involved outdoor presence. In parallel to testing the effect of lockdown measures on avian trait variation, we also analysed the role of tree cover in the nestbox surroundings – a considerably less labile attribute of the urban space than human presence, and vital in providing shelter and food resources (*i.e.* caterpillars as favourite prey item (Perrins 2008)) in these two species.

Based on previous work on human presence carried out in the same study system (Corsini et al. 2017; Seress et al. 2021), we also used the unique opportunity given by the *Anthropause* to test whether human presence *per se* influences the probability of nestbox occupancy, or the earliest stages of reproduction in these two urban adapters. Conversely, we predicted that the percentage of tree cover in nestbox surroundings would maintain its association with great tit and blue tit life-history trait variation, regardless of the pandemic.

## Methods

### Study sites and lockdown restrictions in Poland

**Fig. 1:**
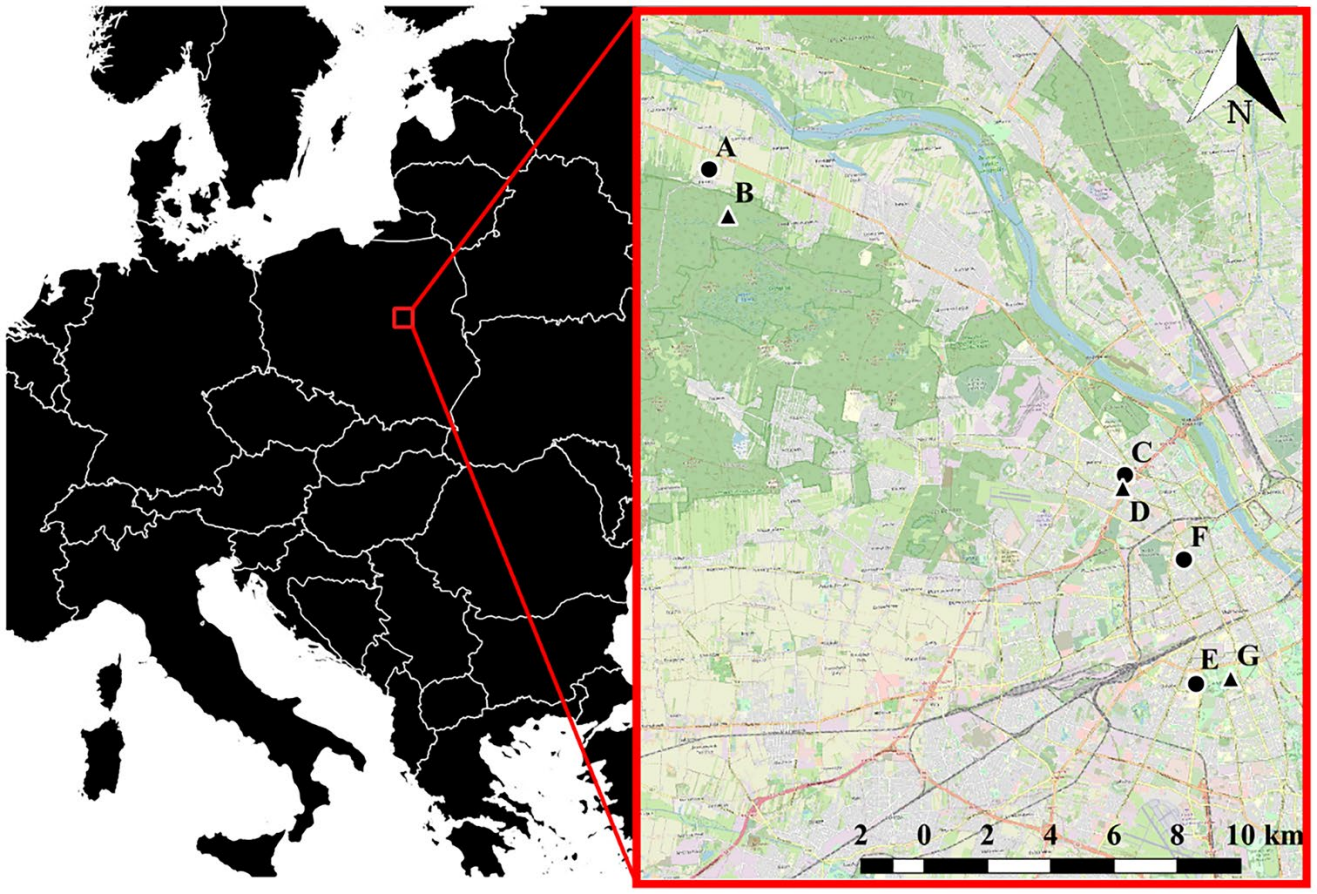
Map of site locations in the Warsaw gradient of urbanisation, Poland. These include: a suburban village (**A**), a natural forest (**B**), two residential areas (**C** and **F**), an urban woodland (**D**), an office area (**E**), and an urban park (**G**). Circles and triangles indicate whether study sites were categorised as “Lockdown – Entrance Allowed (LEA)” or “Lockdown – Entrance Not Allowed (LENA)” sites during the 2020 SARS-CoV-2 pandemic, respectively.

Avian life-history and reproductive data were collected from 2017 to 2020 across seven study sites set in a gradient of urbanisation in the capital city of Warsaw, Poland (Fig. 1). Each study site is characterised by an assigned number of Schwegler woodcrete nestboxes (type 1b, with a 32 mm entrance hole, erected in a 50 m-distance grid and hung in a range of 214–325 cm above the ground) suitable for great tits and blue tits. The study system here described aims at accurately reflecting the urban matrix, as it comprises a wide range of diverse and contrasted habitats (Szulkin et al. 2020b).

While a state of epidemic was officially declared in Poland on March 20^th^, a series of increasingly restrictive measures limiting human presence outdoors was subsequently introduced. A strict lockdown period forbidding the use of urban green areas was introduced from the 1^st^ of April to the 20^th^ of April included. The only activities that could be performed by urban dwellers outside of homes during the period of strict lockdown included the purchase of food supplies and other essentials items, caring duties and work. During this time, access to urban green areas, recreational locations, natural reserves or protected areas within and outside city borders was banned for the general public. However, the authors of this study could access all sites within the remit of their work.

Study areas in our urban study system were subjected to contrasted access restrictions by urban dwellers during the SARS-CoV-2 lockdown:

- “Lockdown - Entrance Allowed” (LEA) – pertains to 4 sites and a total of 173 nestboxes. These included streets and residential areas where residents were allowed outdoors to fulfil their essential needs during the pandemic.
- “Lockdown - Entrance Not Allowed” (LENA) – pertains to 3 sites and a total of 236 nestboxes. These included urban parks, woodlands, national parks and forest reserves, all of which were closed to the public during the strict lockdown period. All these sites re-opened to the public on the 20^th^ of April 2020.

We provide below a brief description of all seven study sites and their associated levels of access restrictions during the pandemic (e.g., “LEA” or “LENA” sites); sites are listed from the most distant to the closest to Warsaw city centre. More details on each study site can be found in Corsini et al. (2017, 2019) and Szulkin et al. (2020b).

(A) **Suburban village (n = 47 nestboxes, LEA).** Palmiry village (20º46′48.9748’’E—52º22′11.3382’’N) is located c. 20 km away from Warsaw city centre and borders Kampinos National Park (Site B). Palmiry is a typical suburban village, where residential homes with gardens are interconnected by tree-lined avenues.
(B) **Natural forest (n = 110, LENA).** Kampinos National Park (20º47′14.3867’’E—52º21′22.5409’’N) is a large forest located c. 20 km from Warsaw city center. The area is characterised by pine and mixed oak-pine forest habitats.
(C) **Residential area II (n = 52, LEA).** Osiedle Olszyna neighbourhood (20º57′39.37097’’E—52º16′23.71883’’N) is characterized by blocks of flats intermixed with green spaces and recreational facilities. It borders with the urban woodland “Las Olszyna” (site D).
(D) **Urban woodland (n = 21, LENA).** Las Olszyna (20º57′33.93652’’E—52º16′10.55093’’N) is a green space that includes a deciduous, wet alder forest and an open space with an adjacent playground.
(E) **Office area (n = 28, LEA).** Warsaw University “Ochota” Campus (20º59′8.85224’’E—52º12′43.77676’’N) is located next to the urban park Pole Mokotowskie (site G) and belongs to one of the central districts of the city. Buildings consist of university offices, laboratories and other student facilities.
(F) **Residential area I (n = 46, LEA).** The Muranow neighbourhood (20º59′5.74332’’E—52º14′52.17925’’N) is a residential area, similar in design to Residential area II (site C).
(G) **Urban park (n = 105, LENA).** Pole Mokotowskie (21º0′6.98321’’E—52º12′46.66874’’N) is an extensive urban green area located close to the city center. It is composed of meadows, tree-covered areas and recreational structures (i.e., playgrounds and sport facilities), and provides a centrally-located recreational area for city dwellers.

### Avian life-history traits data collection

From the end of March, we checked nestboxes weekly to identify those occupied by great tits and blue tits. A nestbox was considered as “occupied” when at least one egg was laid on a completed nest. Weekly checks allowed to precisely record the date of the first egg laid (females usually lay 1 egg per day, and laying date is recorded from the 1^st^ of April, corresponding to the value of 1), incubation duration (given in days and calculated as: hatch date – first egg laid date – clutch size – 1, (Cresswell and McCleery 2003) though incubation occasionally starts earlier or later than clutch completion in tits (Monros et al. 1998)) and clutch size (total number of eggs in the nest). Only first broods were included in the analyses (Balen 1973).

### Tree-cover measurements

We measured the percentage of tree cover in a 100 m radius around each nestbox following Szulkin et al. (2020b). Briefly, we downloaded the raster-layer *Tree Cover Density* from Copernicus Land Monitoring Services (https://land.copernicus.eu/ sitemap; Forests/Tree Cover Density); this raster layer is defined as the vertical projection of tree crowns to a horizontal earth’s surface. The tree cover map was generated in 2015 and contained a 20 m-pixel resolution layer. After creating a 100 m radius buffer around each nestbox, we obtained the averaged value of tree cover (in %) at the nestbox level using the function *Zonal Statistics* in qGIS, as described in Szulkin et al. 2020b.

### Statistical analyses

Statistical analyses were performed within the computing environment R (v.3.6.2), and jointly for great and blue tits (except for occupancy tests, where the two species were analysed separately). The parameter “*Species*” was fitted as a covariate in all the other models in order to directly assess species-specific trait variation. Because there was a significant association between percentage of *Tree cover* and *Lockdown status* (LEA vs. LENA, see Table S2), we investigated the effect of  these two variables in separated models as indicated below.

To test associations between avian life-history traits and lockdown restrictions, all analyses were run using *Linear Models* or *Linear Mixed Effects Models*: note that the category *Site* was fitted as random effect in the model only if the inter-group variance (n = 7 levels) was higher than zero, to control for pseudo-replication issues in occupancy analyses and because of site-related differences in avian fitness. All numeric predictors were mean-centred for clarity of parameters estimates. In addition, all interactions were tested and excluded from the models if not significant.

To investigate the effect of lockdown on avian life-history traits, we specifically focused on the interaction between *Year* and *lockdown status* (LEA—Lockdown Entrance Allowed *vs.* LENA—Lockdown Entrance Not Allowed sites), the latter explicitly reflecting a lack of outdoors human activity in LENA sites in 2020.

To model nestbox *occupancy*, we fitted Generalised Linear Mixed Effects Models (GLMMs) with binomial distribution (“*glmer*” function in the R-package “*lme4*” v.1.1–21-(Bates et al. 2014)). A nestbox was considered occupied (1) only if a great tit or a blue tit (analysed separately) was breeding in the nestbox. Nestbox occupancy (0/1) was fitted as binomial-response variable in each model, while the interaction between the two categorical variables *Year* (four levels: 2017, 2018, 2019 and 2020) and *lockdown status (two levels: LEA and LENA)* were fitted as predictors. For the analysis of nestboxes occupancy, we fitted the interaction between the categorical variables *Year* and *Lockdown status* as explanatory variables.

To model variation in egg *Lay date* (the egg laying date of a nest where the first egg was laid on the 1^st^ of April would be coded with the value of 1), we log-transformed the response variable because the residuals of the models were not normally distributed. We consequently fitted a Linear Model (LM) with a Gaussian distribution (“*lm*” function). The same model structure (LM) was used to model incubation variation in days, *Incubation duration*, where we added *Lay date* as explanatory variable to control for the fact that earlier clutches in the season are characterized by longer incubation periods than those started later in the season, and are often larger than those initiated later (Crick et al. 1993).

For *clutch size*, we ran Linear Mixed Effect Models with Gaussian distribution (“*lmer*” function in R) with a model structure analogous to the one indicated above, but fitting the study site as random effect. Incubation duration -in days- was fitted as response variable while the interaction between *Year* and *Lockdown status*, and the continuous-variable *Lay date* (to control for seasonal differences in each breeding event, as incubation duration decreases later in the season (Corsini et al. 2017)) were fitted as predictors.

To investigate the role of habitat quality on nestbox occupancy and avian life-history traits, we used analogous model structures to those described above, but the parameter *Lockdown* was replaced by the continuous variable *Tree cover* (here used as a proxy of optimal natural food resources, e.g. caterpillars, in great tits and blue tits).

## Results

### No evidence for an association between lockdown restrictions and avian life-history traits

**Fig. 2:**
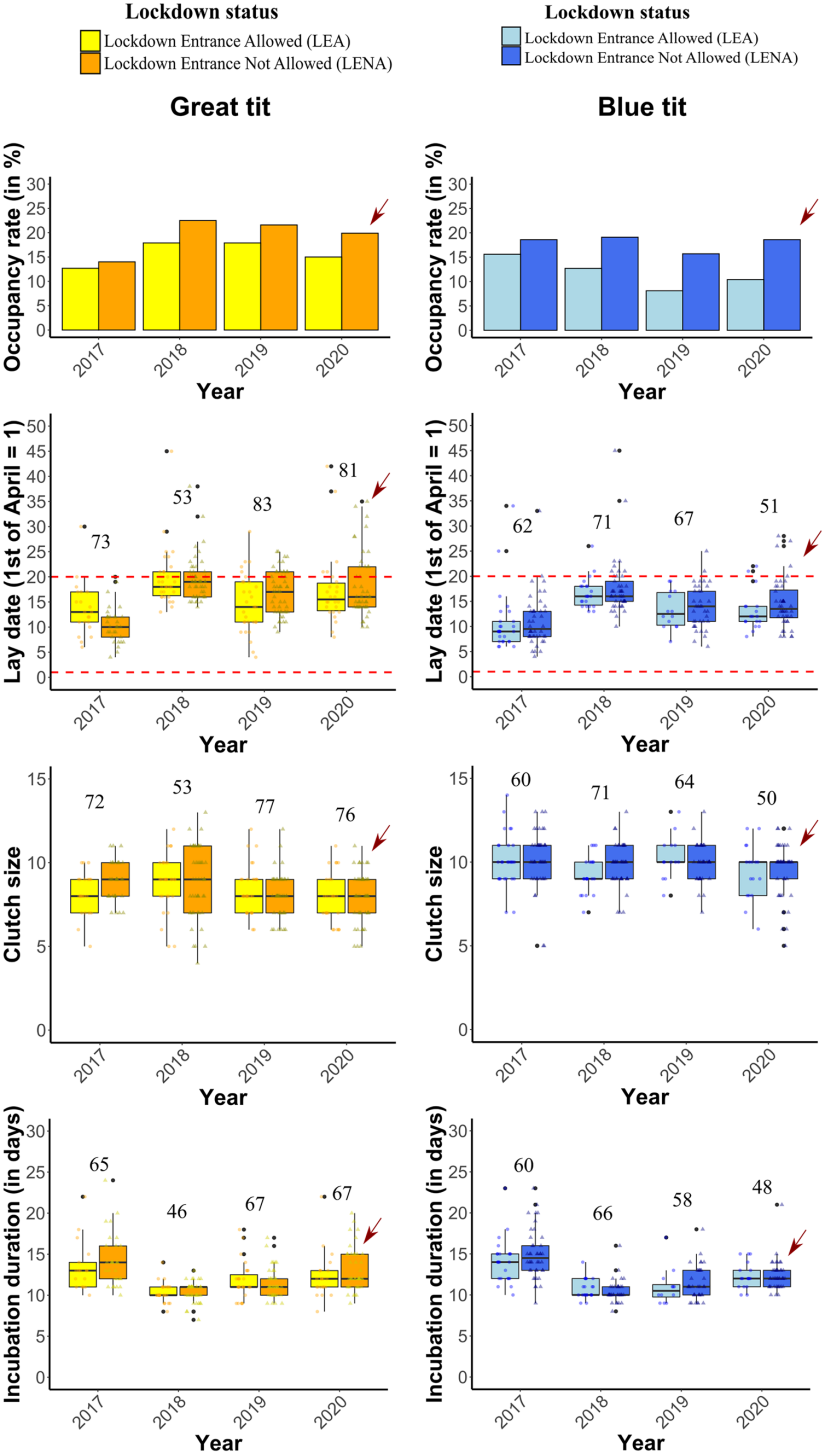
Great tit and blue tit life-history traits comparisons between “Lockdown – Entrance Allowed” sites (LEA, which includes a suburban village, residential and office areas) and “Lockdown – Entrance Not Allowed” sites (LENA, which includes green areas, urban parks and natural reserves) across four years of study. No significant differences between LEA and LENA sites were reported in terms of nestbox occupancy, lay date, clutch size and incubation duration in either of the two species. The red arrow indicates the reference year (2020) and study sites where different lockdown measures were applied (pandemic year -2020- and LENA, respectively). The red-dashed line in lay date graphs indicates the duration of the strictest lockdown restrictions implemented in Poland [from the 1^st^ of April until the 20^th^ of April 2020], mainly focused on forbidding visitor access to urban parks, woodlands and other recreational areas. Dots indicates single observations (nests) grouped by lockdown status (LEA vs LENA); year and species-specific sample sizes are provided above each set of boxplots

There was no significant association between pandemic-related human activity (tested as the interaction Lockdown status*Year) and nestbox occupancy or any of the life-history traits measured (Fig. 2, Tables S3a-d). In contrast, we observed considerable species-specific differences in *Clutch size* and *Lay date*, as well as variation in traits between years. *Lay date* was also negatively associated with clutch size and incubation duration in both great tits and blue tits. In other words, while there was no evidence of a pandemic effect – here studied as the absence of humans within certain study sites—on any life-history traits investigated, clutch size was smaller and incubation duration was shorter later in the season in both species, despite considerable year-to-year fluctuations (Fig. 2, Tables S3a-d).

Lay date occurred earlier in 2017 relative to the pandemic year while, in 2018, lay date trends occurred later relative to the pandemic year (Table S3b). Again, the *Lockdown* status*Year interaction was not significant, we therefore removed it from the model (Table S3b). Incubation duration, which decreased significantly later in the season, was shorter in 2018, 2019 relative to 2020 (Table S3d). Moreover, incubation duration lasted longer in 2017 relative to 2020 (Table S3d).

### Tree cover as driver of probability of nestboxes occupancy and clutch size in great and blue tits

**Fig. 3:**
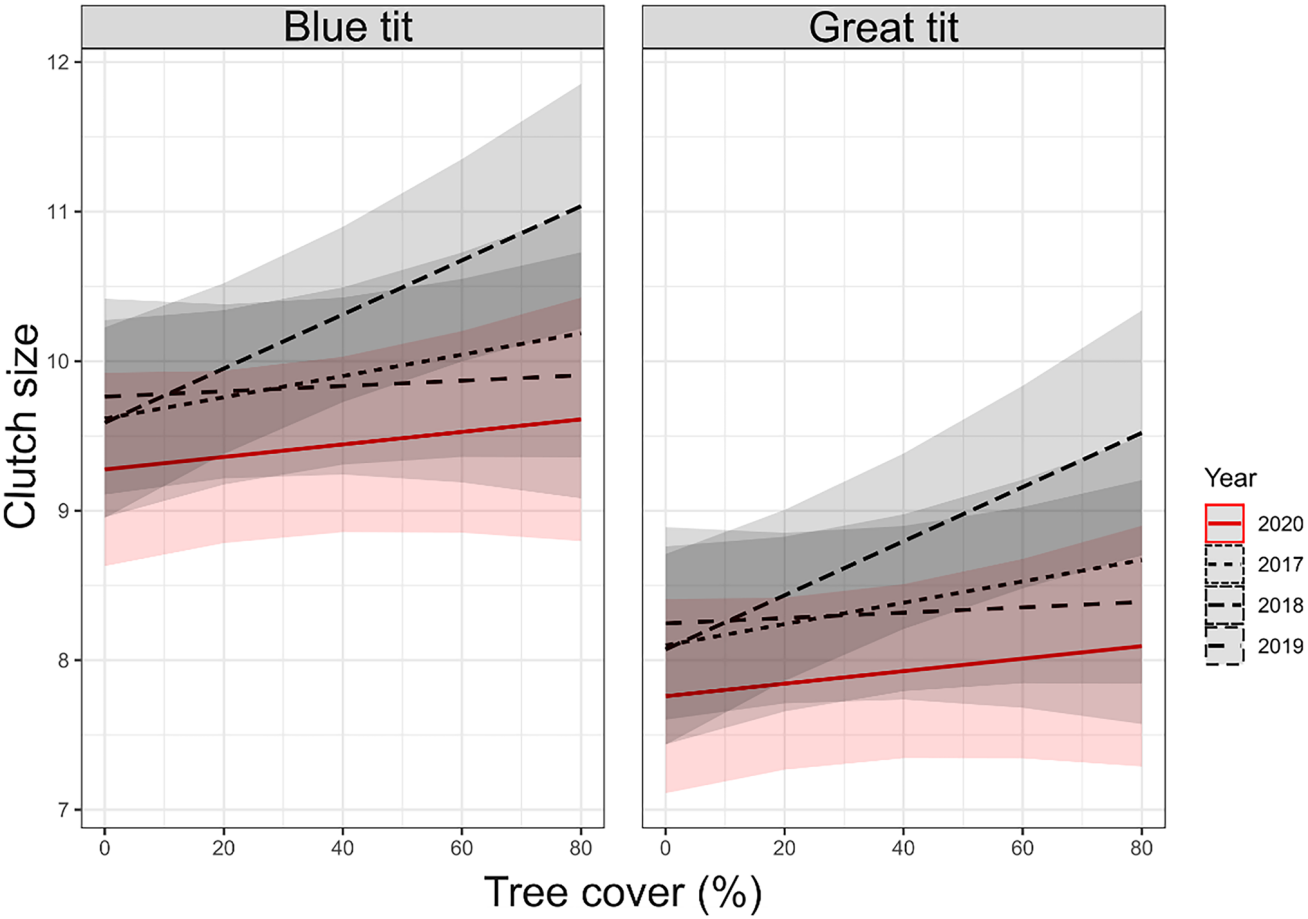
Positive association between tree cover and clutch size in great and blue tits. Linear model (LM) with 95% confidence intervals derived from the *predict* function and visualized in ggplot2. In great tits, the final model included the interaction term *Year* (n = 4 levels, from 2017 to 2020) and *Tree cover*. In blue tits, the final model only included *Tree cover*. Note that in blue tits we included the *Year* partitioning to make the two graphs comparable. In both species, *Lay date* and *Study site* were kept in the final models as fixed and random effects, respectively (see Table S4c for global and final models estimates on mean-centred predictors)

In both great tits and blue tits, tree cover around the nestbox positively covaried with the probability of nestbox occupancy (Table 1). Tree cover was also positively associated with lay date in great tits (clutches in areas with a larger percentage of tree cover were initiated later in the season), and clutch size in blue tits and great tits (Table S4a, Fig. 3; note significant Tree cover*Year interaction for great tits). In line with previous work, incubation duration decreased later in the season (Table S4c). Importantly, for all traits but clutch size, the Tree cover * Year interaction was not significant and was thereforeremoved from the final models (Table S4a-c).

**Table 1:** The probability of nestbox occupancy was positively associated with increased % of tree cover in the nestbox surroundings. Generalised Linear Mixed Effects Models (GLMMs) testing the association between the probability of nestbox occupancy (with binomial distribution) and tree cover in great and blue tits. *Tree cover* was mean-centred for clarity of parameter estimation. Study site was fitted as random effect

**Occupancy and Tree cover (%)**										
**Great tit**						**Blue tit**				
Global model: Occupancy (1/0) ~ Tree cover_scaled-cent_ * Year, Random = Site (n = 7). *Family* = binomial						Global model: Occupancy (1/0) ~ Tree cover_scaled-cent_ * Year, Random = Site (n = 7). *Family* = binomial				
**Variable**	**estimate**	**se**	**z-value**	**p-value**		**Variable**	**estimate**	**se**	**z-value**	**p-value**
Intercept	-1.500	0.240	-6.250	<0.001***		Intercept	-1.663	0.325	-5.110	< 0.001***
**Tree cover**_sc_	**0.023**	**0.007**	**3.430**	<0.001***		**Tree cover**_**sc**_	**0.016**	**0.007**	**2.240**	0.025*
Year 2017	-0.323	0.199	-1.630	0.104		Year 2017	0.184	0.196	0.940	0.349
Year 2018	0.207	0.182	1.140	0.254		Year 2018	0.109	0.198	0.550	0.581
Year 2019	0.175	0.183	0.960	0.338		Year 2019	-0.228	0.209	-1.090	0.277
n = 1636, (1 = 294, 0 = 1342)						n = 1636, (1 = 251, 0 = 1385)				

Significance levels: *p < 0.05, **p < 0.01, ***p < 0.001

## Discussion

We did not detect any significant Lockdown status*Year interaction on the probability of nestbox occupancy, nor on any other reproductive  life-history traits in two passerine birds breeding in a gradient of urbanisation (specifically lay date, clutch size and incubation duration). These results are in line with the study conducted by Seress et al. ( 2021), who also reported no effect of the covid lockdown on great tit reproductive success (i.e., no effect on clutch size, brood size and fledging success). Our results also supports earlier work carried out in the same study population, reporting no effects of human presence* per se *on avian reproduction in the urban space (Corsini et al. 2017). Here, we found no support for the hypothesis that the 2020 pandemic lockdown – here studied as the lack of humans within certain study areas – was associated with blue tit or great tit life-history reproductive variation (Fig. 2, Tables S3a-d). Instead, occupancy and life-history traits in great tits and blue tits were most likely subjected to a strong inter-annual variation caused by other biotic and abiotic factors. In contrast to a lack of effects of human presence, we established that tree cover was positively associated with the probability of nestbox occupancy and clutch size in both species (Table 1, Fig. 3). Most importantly, there was a significant effect of the interaction between *tree cover* and *year* on clutch size. The most likely proximate factors underlying this relationship are tree-dwelling lepidopteran larvae and other insects, which remain the main food items found on tree canopy, and are used by adult tits to feed their offspring in natural and urban-dominated landscapes (Perrins 2008) (but see Pollock et al. 2017; Sinkovics et al. 2021, reporting that blue tits and great tits may also occasionally use human-generated food resources to feed their nestlings). Importantly, the year- dependent effect of tree cover underlines the importance of inter-annual variation in prey availability (e.g., rainy vs. dry years, see work by Morganti et al. 2017). Overall, tree cover is likely to act as cue for resource allocation in reproductive decisions.

Here, we used the *Anthropause* (Rutz et al. 2020) triggered by the SARS-CoV-2 pandemic, and set in the context of avian breeding data collected across multiple years in a heterogeneous urban landscape, to confirm the limited role of human presence in avian reproductive decisions using the unique opportunity furnished by this “quasi” experimental approach. Interestingly, bird preferences in terms of the extent of tree cover surrounding the nestboxes did not change in the pandemic year relative to previous years. As humans disappeared for 20 days from LENA sites in 2020, these unique circumstances could have prompted birds to occupy nestboxes more evenly relative to earlier years, as human abundances covary positively with low levels of tree cover in the urban space (Szulkin et al. 2020b). This may suggest that great tits and blue tits are selecting their breeding locations irrespective of human presence* per se*, and that the ecological cues such as tree cover used by these two urban adapters remain unchanged in their reproductive decisions, regardless of the *Anthropause* (Fig. 2, Fig. 3).

It is now well-established that the SARS-CoV-2 pandemic lockdown generated a large range of biological responses across several *taxa –* from no response to considerable positive and negative deviations from original trait distributions recorded in the years preceding the pandemic (Table S1 and review by Bates et al. 2021). This is particularly evident in the available works investigating phenotype or fitness data in avian populations breeding within urban environments (Seress et al. 2021; Manenti et al. 2020). Aside for the lack of lockdown effects in blue tits and great tits reported here, and a lack of effects on great tits reported by Seress et al. ( 2021), Manenti et al. (2020) recorded an increase in clutch size in the common swift in Italy in the pandemic year. The authors attribute this positive trend in reproductive success to the drastic drop of air pollution recorded in 2020 in the country (specifically, nitrogen dioxide, benzene and sulphur dioxide (Campbell and Vallano 2018). In fact, nitrogen dioxide reduces insect biomass but may also directly affect birds fitness by inhalation exposure (Campbell and Vallano 2018; Sanderfoot and Holloway 2017). Differently to tits -which feed on canopy invertebrates during the breeding season (Perrins 2008; Sinkovics et al. 2021)- common swifts are aerial Afro-Palearctic migratory birds, which exclusively feed on aerial insects (Margerie et al. 2018). Therefore, swifts may be more sensitive than tits to changes in aerial insect abundance in the urban space, though this relationship remains speculative. This is also supported by the study conducted by Seress et al., where the drop in air pollution levels recorded during the lockdown restrictions was not associated with great tit breeding success (Seress et al. 2021). Other studies carried out on avian breeding success in the *Anthropause* (Table S1) highlighted the role of food availability in species occurrence (Soh et al. 2021; Gilby et al. 2021). In fact, species like Feral pigeons (*Columba livia*) and Torresian crows (*Corvus orru*) (often referred to as *urban exploiters* as they rely on waste, bread, seeds and other anthropogenic food resources (Palacio 2020; Gilby et al. 2021)) decreased in number within the urban space since the pandemic started, moving to more natural areas to feed on native invertebrate communities, with serious (and, to some extent, irreversible) consequences on local ecosystems (Gilby et al. 2021). Therefore, the results described above (and summarised in Table S1) emphasise a strong species-specific response to lockdown restrictions in avian communities.

Another possible reason for species-specific responses to the covid-triggered lockdown in birds may be related to their breeding preferences. For example, great tits and blue tits are cavity-nesters and as such, are not directly exposed to humans or dogs (which are often associated with human presence in urban areas, see Corsini et al.(2019)) during the breeding season. It is possible that data from open and/or ground-nesting birds could reveal a more complex picture of lockdown-effects associated with human presence on avian breeding success (see Hentati-Sundberg et al. (2021)). Such effects driven by predation, however, remain difficult to disentangle and quantify: while human presence may expose certain ground and open nesting birds to predation by dogs—as they are often left free to roam in urban parks, it may also reduce the activity of other predators both directly and indirectly (see review by Vincze et al. (2017)).

While the results presented in this study support earlier work conducted in the same study set-up (Corsini et al. 2017), as well as findings described by Seress et al. (2021), we here highlight the overarching role of year-effects detected in most of the great tit and blue tit life-history traits examined. Indeed, the role of inter-annual variation on reproductive success has been readily described in natural and urban populations of great and blue tits (Glądalski 2015; Marciniak et al. 2007). For example, caterpillar biomass is strongly associated with temperature and rainfall (Schöll et al. 2016). In addition, years characterized by extreme climatic conditions may directly cause brood reduction and nest failure, as shown with heavy and persistent rainfall (Morganti et al. 2017; Schöll and Hille 2020). Also, Dhondt et al. (1992), reported that tits may increase clutch size in relation to habitat quality, and such differences was manifested from one year to another. Consequently, inter-annual variation driven by both abiotic and biotic factors may further complicate the disentangling of year effects from “one-off” events of interest, such as the occurrence of lockdown restrictions. Moreover, lockdown restrictions and here presented occurred only once and cannot be repeated on multiple “treatment” years. All in all, we argue that these are important limitations that must be taken into account while interpreting most of the studies ran during the pandemic (Table S1).

Undoubtedly, lockdown timing, combined with species biology related to diet, behaviour or breeding preferences, might have played a decisive role in the contrasted fitness responses recorded during the pandemic (Table S1). More studies would be valuable to reliably explore the fine-scale dynamics between human presence and wildlife biological variation in the *Anthropause*.

## Conclusions

The short-term restrictions imposed by the SARS-CoV-2 pandemic lockdown were not associated with nestbox occupancy, nor any of the reproductive life-history traits measured in great tits and blue tits in an Eastern European capital city (Warsaw, Poland). Our results, combined with those reported by recent studies carried out during the pandemic (Table S1), point to a complex picture of lockdown consequences on urban wildlife, which are likely to be species-specific (e.g., related to diet or reproductive strategies), and context-related (e.g., dependent on the location and timing of lockdown or of human responses to it).

We argue that the use of longer-term studies (reporting > 2 years of breeding data) in the specific context of the “*Anthropause*” generated by the SARS-CoV-2 pandemic would reveal a fuller and more balanced picture of the diverse urban wildlife responses. Ultimately, such studies would pave the way to a better understanding of rapid life-history and behavioural responses of wildlife to human activities – or to their drastic reduction, as observed during the SARS-CoV-2 pandemic.

## Supplementary Information

Below is the link to the electronic supplementary material.

Supplementary file1 (DOCX 72.6 KB)

## Data Availability

Data are available on Dryad Digital Repository (Corsini et al., 2022).

## Code availability

Custom code available via request to authors. QGIS, R and Rstudio are software applications freely available to download.
