# Supplementary material for: Breeding in the pandemic: short-term lockdown restrictions in a European capital city did not alter the life-history traits of two urban adapters
Source: Urban Ecosyst. 2022 Dec 14:1–11. Online ahead of print. doi: 10.1007/s11252-022-01309-5 (PMC9748896; doi:10.1007/s11252-022-01309-5)
Supplement: Supplementary file 1 — Supplementary file1 (DOCX 72.6 KB) [file 11252_2022_1309_MOESM1_ESM.docx]

**Supplementary Information**

**Breeding in the pandemic: short-term lockdown restrictions in a European capital city did not alter the life-history traits of two urban adapters**

**Michela Corsini ^1^*, Zuzanna Jagiello^1,2^, Michał Walesiak^1,3^, Michał Redlisiak^1,4^, Ignacy Stadnicki^1,5^, Ewa Mierzejewska^1^ & Marta Szulkin^1^**

^1^ Centre of New Technologies, University of Warsaw, ul. Banacha 2c, 02-097 Warsaw, Poland.

^2^ Department of Zoology, Poznań University of Life Sciences, Wojska Polskiego 71C, 60-625 Poznań, Poland.

^3^ Mammal Research Institute, Polish Academy of Sciences, ul. Stoczek 1, 17-230 Białowieża, Poland.

^4^ Faculty of Biology, University of Gdansk, Bird Migration Research Station, ul. Wita Stwosza 59, 80-308, Gdansk, Poland.

^5^ Artes Liberales, University of Warsaw, ul. Nowy Świat 69, 00-046 Warsaw, Poland.

*corresponding author: e-mail: [michela.corsini.fau@gmail.com](mailto:michela.corsini.fau@gmail.com)

**Abstract**

Humans are transforming natural habitats into managed urban green areas and impervious surfaces at an unprecedented pace. Yet the effects of human presence *per se* on animal life-history traits are rarely tested. This is particularly true in cities, where human presence is often indissociable from urbanisation itself. The onset of the SARS-CoV-2 outbreak, along with the resulting lockdown restrictions, offered a unique, “natural experiment” context to investigate wildlife responses to a sudden reduction of human activities. We analysed four years of avian breeding data collected in a European capital city to test whether lockdown measures altered nestbox occupancy and life-history traits in terms of egg laying date, incubation duration and clutch size in two urban adapters: great tits (*Parus major*) and blue tits (*Cyanistes caeruleus*). Lockdown measures, which modulated human presence, did not influence any of the life-history traits investigated. In contrast, tree cover, a distinct ecological attribute of the urban space, was positively associated with clutch size, a key avian life-history and reproductive trait. This highlights the importance of habitat quality over human activity on reproduction of urban wildlife. We discuss our results in light of other urban wildlife studies carried out during the pandemic, inviting the scientific community to carefully interpret all lockdown - associated shifts in biological traits.

***Keywords:*** lockdown, SARS-CoV-2, urbanisation, human presence, avian life-history traits, tree cover

| **Study species** | **Traits** | **Human action** | **Behaviour** | **Effect^b^** | **Study area / habitat type** | **Methods^c^** | **Lockdown period^d^** | **Ref.** |
| --- | --- | --- | --- | --- | --- | --- | --- | --- |
| Greater snow goose |  | Scaring events |  | (-) | Small agricultural island | FW | 16^th^ of March – 4^th^ of May 2020 | ^1^ |
| (*Chen caerulescens atlantica*) | Body condition |  |  | (+) | Saint-Lawrence estuary | FW |  |  |
|  |  | Hunting activities |  | (-) | [Canada] | FW |  |  |
| Avian species |  |  | Presence | (nd)  (+) | Urban and rural areas [north eastern Spain]  Urban and rural areas [north eastern Spain] | CZS | 15^th^ of March – 13^th^ of April 2020 | ^2^ |
|  |  |  | Morning detectability |  |  | CZS |  |  |
| Avian species |  | Submission of protocol lists to SABAP2 |  | (-) | [South Africa] | CZS | 27^th^ of March – 30^th^ of April 2020 | ^3^ |
|  |  | Participation to “Lockdown-gardens” surveys |  | (+) |  | CZS |  |  |
| Eurasian tree sparrow (*Passer montanus*) |  |  | Flight Initiation Distance | (-) | Urban areas [China] | FW | [ns] | ^4^ |
| Puma (*Puma concolor*) |  |  | Presence | (+) | [North American urban centres] | CZS | [1^st^ of March – 31^st^ of July 2020] | ^5^ |
| Coyote (*Canis latrans*), |  |  | Presence | (nd) | [North American urban centres] | CZS |  |  |
| Moose (*Alces alces*) |  |  | Presence | (nd) | [North American urban centres] | CZS |  |  |
| American black bear (*Ursus americanus*) |  |  | Presence | (nd) | [North American urban centres] | CZS |  |  |
| Bobcat (*Lynx rufus*) |  |  | Presence | (nd) | [North American urban centres] | CZS |  |  |
| Feral pigeons (*Columba livia*) |  |  | Presence | (-) | Urban - open food centres - Singapore | FW | 7^th^ of April – 1^st^ of June 2020 | ^6^ |
| Feral pigeons (*Columba livia*) |  |  | Foraging and moving | (+) | Urban – foraging hotspots - Singapore | FW |  |  |
| Feral pigeons (*Columba livia*) |  |  | Resting | (-) | Urban – foraging hotspots - Singapore | FW |  |  |
| Javan myna (*Acridotheres javanicus*) |  |  | Presence | (+) | Urban – refuse collection centre - Singapore | FW |  |  |
| Javan myna (*Acridotheres javanicus*) |  |  | Presence | (-) | Urban – green areas - Singapore | FW |  |  |
| Common myna (*Acridotheres tristis*) |  |  | Presence | (nd) | Urban - open food centres - Singapore | FW |  |  |
| House crow (*Corvus splendens*) |  |  | Presence | (nd) | Urban – foraging hotspots - Singapore | FW |  |  |
| Torresian crows (*Corvus orru*) |  |  | Presence | (-)  (+) | Urban  Rural / natural | FW | 2^nd^ of April – 30th of June 2020 | ^7^ |
|  |  |  | Presence |  |  | FW |  |  |
| White crowned sparrow |  |  | Amplitude of song | (-) | San Francisco Bay [United States of America] | FW | 1^st^ of April – 31^st^ of May 2020 | ^8^ |
| *(Zonotrichia leucophrys*) |  | Noise levels |  | (-) | San Francisco Bay [United States of America] | FW |  |  |
|  |  |  | Song performance | (+) | San Francisco Bay [United States of America] | FW |  |  |
| Leatherback sea turtle |  |  | Presence | (+) | Beach on the North Caribbean coast [Costa Rica] | FW | 1^st^ of March – 31^st^ of August 2020 | ^9^ |
| (*Dermochelys coriacea*) | Hatching success |  |  | (+) | Beach on the North Caribbean coast [Costa Rica] | FW |  |  |
| Crested porcupine (*Hystrix cristata*) |  |  | Presence | (+) / (nd) | Urban areas / non – urban areas [Italy] | CZS | 11^th^ of March – 4^th^ of May 2020 | ^10^ |
| Kentish plover (*Charadrius alexandrinus*) |  |  | Presence | (+) | Seaside [Italy] | FW |  |  |
| Water birds |  |  | Species richness | (+) | Artificial lake [northern Italy] | FW |  |  |
| Common swift (*Apus apus*) | Clutch size |  |  | (+) | [Italy] | FW |  |  |
| Common toads (*Bufo bufo*) |  | Road kill |  | (-) | [Italy] | FW |  |  |
| Agile frog (*Rana dalmatina*) |  | Road kill |  | (-) | [Italy] | FW |  |  |
| Common wall lizard (*Podarcis muralis*) |  | Road kill |  | (-) | [Italy] | FW |  |  |
| Western green lizard (*Lacerta bilineata*) |  | Road kill |  | (-) | [Italy] | FW |  |  |
| Eastern cottontail (*Sylvilagus floridanus*) |  |  | Diurnal activity | (+) | [northern Italy] | FW |  |  |
| Wildlife |  | Poaching activities |  | (+) | Protected areas [Nepal] | FW | 24^th^ of March – 21^st^ of June 2020 | ^11^ |
|  |  |  | Presence | (+) | Protected areas [Nepal] | FW |  |  |
| Bats species |  | Negative attitude from the public |  | (+) | [China] | CZS | [ns] | ^12^ |
| Hedgehog (*Erinaceus roumanicus*) |  | Road kill |  | (-) | Chełm [Poland] | FW | [ns] | ^13^ |
| Great tit (*Parus major*) | Nestlings body mass  Clutch size, hatching success, Nr of nestlings,  Nr of fledglings  clutch size, hatching success, Nr of nestlings, Nr of fledglings, nestlings body mass |  |  | (-)  (nd)  (nd)  (nd)  (nd) | Budapest [Hungary]  Budapest  Budapest  Veszprém [Hungary]  Veszprém | FW | [ns] | ^14^ |

**Table S1.** Overview of the earliest studies available testing the influence of lockdown restrictions on wildlife. Papers search was conducted using both Google Scholar and Web of Science, and included research papers published between March 2020 and September 2021. Papers here indicated analysed the lockdown effects on wildlife. Column (^a^) categorises behavioural responses (note that here, “behaviour” is not qualified as phenotype, as this category is largely constituted of species sightings rather than individual-specific attributes), individual-level phenotype and fitness (Traits) or to wildlife-related human activities (Human actions). Effects (^b^) report the directionality of the association driven by the lockdown period, denoted as an increase (+), decrease (-) or no significant difference (nd). Methods of data collection detailed in (^c^) categorise data collection as performed by scientists during fieldwork (FW) or recorded as contributions of volunteers (CZS, a citizen science approach), which recorded daily-based observations through online or other social media platforms. (^d^) indicates the length of the lockdown period, where “ns” indicates “not specified” in the study).

| **Species** | **Mean % tree cover ± se by lockdown status** | **One-Way ANOVA** |
| --- | --- | --- |
| Great tit (n = 231) | Lockdown _LEA_ = 9.17 ±1.14 (n = 108),  Lockdown _LENA_ = 49.4 ±1.91 (n = 183) | F (1, 289) = 231, p < 0.001*** |
| Blue tit (n = 251) | Lockdown _LEA_ = 6.87 ±1.05 (n = 81),  Lockdown _LENA_ = 40.6 ±1.78 (n = 170) | F (1, 249) = 158, p < 0.001*** |

**Table S2. One-Way ANOVAs testing the association between percentage tree cover and Lockdown status in great tits and blue tits**. Lockdown status includes LENA (areas where Lockdown Entrance was Not Allowed to visitors in 2020. This category included green areas such as urban parks, recreational sites as well as natural reserves), and LEA study sites (study sites where Lockdown Entrance was always allowed, regardless of lockdown restrictions. This category included residential and office sites and a suburban village). **Significance levels: *p < 0.05, **p < 0.01, ***p < 0.001.**

| **Occupancy and Lockdown restrictions** | | | | | | | | | | |
| --- | --- | --- | --- | --- | --- | --- | --- | --- | --- | --- |
| **Great tit** | | | | |  | **Blue tit** | | | | |
| Global model: Occupancy (1/0) ~ Lockdown status + Year  *Family* = binomial, Random = Site (n = 7) | | | | |  | Global model: Occupancy (1/0) ~ Lockdown status + Year  *Family* = binomial, Random = Site (n = 7) | | | | |
| **Variable** | **estimate** | **se** | **z-value** | **p-value** |  | **Variable** | **estimate** | **se** | **z-value** | **p-value** |
| Intercept | -1.758 | 0.265 | -6.620 | <0.001*** |  | Intercept | -2.158 | 0.337 | -6.390 | <0.001*** |
| Lockdown LENA | 0.326 | 0.364 | 0.900 | 0.370 |  | Lockdown LENA | 0.698 | 0.465 | 1.500 | 0.130 |
| Year 2017 | -0.197 | 0.314 | -0.630 | 0.530 |  | Year 2017 | 0.470 | 0.325 | 1.440 | 0.150 |
| Year 2018 | 0.215 | 0.293 | 0.730 | 0.460 |  | Year 2018 | 0.228 | 0.338 | 0.680 | 0.500 |
| Year 2019 | 0.215 | 0.293 | 0.730 | 0.460 |  | Year 2019 | -0.279 | 0.373 | -0.750 | 0.450 |
|  |  |  |  |  |  |  |  |  |  |  |
| n = 1636, (1 = 294, 0 = 1342) | | | | |  | n = 1636, (1 = 251, 0 = 1385) | | | | |

**Table S3a.** **Generalised Linear Mixed Effects Models (GLMMs) testing the association between probability of nestbox occupancy and lockdown restrictions in great and blue tits. Species were tested separately.** Probability of nestboxes occupancy was fitted using a binomial distribution (0 = as not occupied or occupied by other species than tits, 1= as occupied by great or blue tits). Study site was fitted as a random effect to avoid pseudo-replication issues. 2020, the pandemic year, was used as reference. Study site was fitted as random effect in each model. **Significance levels are reported in bold: *p < 0.05, **p < 0.01, ***p < 0.001.**

**Lay date and Lockdown restrictions**

| **Variable** | **Estimate** | **se** | **t-value** | **p-value** |
| --- | --- | --- | --- | --- |
| (Intercept) | 2.612 | 0.054 | 48.510 | <0.001*** |
| **Species (Great tit)** | **0.154** | **0.028** | **5.460** | **<0.001***** |
| Lockdown (LENA) | 0.036 | 0.029 | 1.240 | 0.216 |
| Year |  |  |  |  |
| **Year 2017** | **-0.358** | **0.040** | **-8.860** | **<0.001***** |
| **Year 2018** | **0.174** | **0.038** | **4.530** | **<0.001***** |
| Year 2019 | -0.066 | 0.039 | -1.660 | 0.098 |
| n = 541 |  |  |  |  |

**Table S3b. Linear models testing the association between Lay date and lockdown status (LENA: Lockdown Entrance Not Allowed).** Lay date (1 = 1^st^ of April) was log-transformed and fitted as response. 2020, the pandemic year, was used as reference. **Significance levels: *p < 0.05, **p < 0.01, ***p < 0.001.**

**Clutch size and Lockdown restrictions**

| **Variable** | **Estimate** | **se** | **t-value** | **p-value** |
| --- | --- | --- | --- | --- |
| (Intercept) | 9.135 | 0.443 | 20.620 | <0.001*** |
| **Lay date _sc_** | -**0.058** | **0.012** | **-5.040** | **<0.001***** |
| **Species (Great tit)** | -**1.520** | **0.124** | **-12.290** | **<0.001***** |
| Lockdown (LENA) | 0.446 | 0.644 | 0.690 | 0.520 |
| **Year** |  |  |  |  |
| **Year 2017** | **0.449** | **0.177** | **2.530** | **0.012*** |
| **Year 2018** | **0.759** | **0.166** | **4.580** | **<0.001***** |
| **Year 2019** | **0.418** | **0.167** | **2.490** | **0.013*** |
| n = 523 |  |  |  |  |

**Table S3c. Linear Mixed Effect Model (LMMs) testing the association between clutch size and lockdown restrictions.** Clutch size was modelled with a Gaussian distribution. Study site was fitted as a random effect (7 levels). 2020, the pandemic year, was used as reference. Lay date (recorded as 1 = 1^st^ of April each year) was mean-centered for clarity of parameters’ estimates. **Significance levels are reported in bold: *p < 0.05, **p < 0.01, ***p < 0.001.**

**Incubation duration and Lockdown restrictions**

| **Variable** | **Estimate** | **se** | **t-value** | **p-value** |
| --- | --- | --- | --- | --- |
| (Intercept) | 2.490 | 0.020 | 120.880 | <0.001*** |
| **Lay date _sc_** | **-0.008** | **0.002** | **-5.770** | **<0.001***** |
| Species (Great tit) | 0.021 | 0.016 | 1.360 | 0.174 |
| Lockdown (LENA) | 0.029 | 0.016 | 1.780 | 0.075 |
| **Year** |  |  |  |  |
| **Year 2017** | **0.101** | **0.022** | **4.480** | **<0.001***** |
| **Year 2018** | **-0.149** | **0.021** | **-6.930** | **<0.001***** |
| **Year 2019** | **-0.096** | **0.021** | **-4.470** | **<0.001***** |
| n = 477 |  |  |  |  |

|  |
| --- |

**Table S3d. Linear Models (LM) testing the association between incubation duration (in days) and lockdown restrictions.** Incubation duration (in days, calculated following Cresswell and Mccleery, ^15^) was log-transformed and modelled with a Gaussian distribution. 2020, the pandemic year, was used as reference. Lay date (recorded as 1 = 1^st^ of April each year) was mean-centered for clarity of parameters’ estimates. **Significance levels are reported in bold: *p < 0.05, **p < 0.01, ***p < 0.001.**

**Lay date and tree cover**

| **Variable** | **Estimate** | **se** | **t-value** | **p-value** |
| --- | --- | --- | --- | --- |
| (Intercept) | 2.640 | 0.031 | 84.160 | <0.001*** |
| **Species (Great tit)** | **0.142** | **0.028** | **5.100** | **<0.001***** |
| Tree cover _sc_ | **0.002** | **0.0005** | **3.790** | **<0.001***** |
| Year |  |  |  |  |
| **Year 2017** | **-0.360** | **0.039** | **-9.030** | **<0.001***** |
| **Year 2018** | **0.177** | **0.038** | **4.670** | **<0.001***** |
| Year 2019 | -0.062 | 0.039 | -1.580 | 0.114 |
| n = 541 |  |  |  |  |

**Table S4a. Linear model (LM) testing the association between *Lay date* and *Tree cover*.** Lay date (recorded as 1 = 1^st^ of April) was log-transformed and modelled with a Gaussian distribution. 2020, the pandemic year, was used as reference. Tree cover (measured in a 100m radius around each nestbox) was mean-centered for clarity of parameters’ estimates. **Significance levels are reported in bold: *p < 0.05, **p < 0.01, ***p < 0.001.**

**Clutch size and tree cover**

| **Variable** | **Estimate** | **se** | **df** | **t-value** | **p-value** |
| --- | --- | --- | --- | --- | --- |
| (Intercept) | 9.412 | 0.292 | 8.427 | 32.220 | <0.001*** |
| **Lay date _sc_** | **-0.057** | **0.011** | **508.494** | **-4.990** | **<0.001***** |
| **Species (Great tit)** | **-1.516** | **0.123** | **508.752** | **-12.320** | **<0.001***** |
| Tree cover _sc_ | 0.004 | 0.006 | 305.607 | 0.740 | 0.459 |
| Year |  |  |  |  |  |
| **Year 2017** | **0.435** | **0.176** | **508.280** | **2.470** | **0.014*** |
| **Year 2018** | **0.761** | **0.165** | **507.299** | **4.620** | **<0.001***** |
| **Year 2019** | **0.409** | **0.167** | **507.234** | **2.450** | **0.015*** |
| Tree cover _sc_ * Year |  |  |  |  |  |
| Tree cover_sc_ * 2017 | 0.003 | 0.006 | 507.745 | 0.490 | 0.625 |
| **Tree cover_sc_ * 2018** | **0.014** | **0.006** | **507.270** | **2.370** | **0.018*** |
| Tree cover_sc_ * 2019 | -0.002 | 0.006 | 507.145 | -0.400 | 0.690 |
| n = 523 |  |  |  |  |  |

**Table S4b. Linear Mixed Effects Models (LMMs) testing the association between *clutch size* and *tree cover*.** Clutch size was modelled with a Gaussian distribution. Study site was fitted as a random effect (7 levels) for each model. 2020, the pandemic year, was used as reference. Tree cover (measured in a 100m radius around each nestbox) and lay date (recorded as 1 = 1^st^ of April) were mean-centered for clarity of parameters’ estimates. **Significance levels are reported in bold: *p < 0.05, **p < 0.01, ***p < 0.001.**

**Incubation duration and tree cover**

| **Variable** | **Estimate** | **se** | **t-value** | **p-value** |
| --- | --- | --- | --- | --- |
| (Intercept) | 2.510 | 0.017 | 148.020 | <0.001*** |
| **Lay date _sc_** | **-0.008** | **0.002** | **-5.420** | **<0.001***** |
| Species (Great tit) | 0.020 | 0.016 | 1.300 | 0.190 |
| Tree cover _sc_ | -0.0004 | 0.0003 | -1.250 | 0.210 |
| Year |  |  |  |  |
| **Year 2017** | **0.101** | **0.023** | **4.470** | **<0.001***** |
| **Year 2018** | **-0.155** | **0.022** | **-7.130** | **<0.001***** |
| **Year 2019** | **-0.097** | **0.021** | **-4.510** | **<0.001***** |
| n = 477 |  |  |  |  |

**Table S4c.** **Linear Model (LM) testing the association between incubation and tree cover.** Incubation duration (in days, calculated following Cresswell and Mccleery, ^15^) was log-transformed and fitted as Gaussian response variable. Tree cover (measured in a 100m radius around each nestbox) and lay date (recorded as 1 = 1^st^ of April) were mean-centered for clarity of parameters’ estimates. **Significance levels are reported in bold: *p < 0.05, **p < 0.01, ***p < 0.001.**

**References**

1. LeTourneux, F. *et al.* COVID19-induced reduction in human disturbance enhances fattening of an overabundant goose species. *Biological conservation* **255**, 108968 (2021).

2. Gordo, O., Brotons, L., Herrando, S. & Gargallo, G. Rapid behavioural response of urban birds to COVID-19 lockdown. *Proceedings of the Royal Society B: Biological Sciences* **288**, 20202513 (2021).

3. Rose, S., Suri, J., Brooks, M. & Ryan, P. G. COVID-19 and citizen science: lessons learned from southern Africa. *Ostrich* **91**, 188–191 (2020).

4. Jiang, X., Liu, J., Zhang, C. & Liang, W. Face masks matter: Eurasian tree sparrows show reduced fear responses to people wearing face masks during the COVID-19 pandemic. *Global Ecology and Conservation* **24**, e01277 (2020).

5. Vardi, R., Berger-Tal, O. & Roll, U. iNaturalist insights illuminate COVID-19 effects on large mammals in urban centers. *Biological Conservation* **254**, 108953 (2021).

6. Soh, M. C. K. *et al.* Restricted human activities shift the foraging strategies of feral pigeons (Columba livia) and three other commensal bird species. *Biological Conservation* **253**, 108927 (2021).

7. Gilby, B. L. *et al.* Potentially negative ecological consequences of animal redistribution on beaches during COVID-19 lockdown. *Biological Conservation* **253**, 108926 (2021).

8. Derryberry, E. P., Phillips, J. N., Derryberry, G. E., Blum, M. J. & Luther, D. Singing in a silent spring: Birds respond to a half-century soundscape reversion during the COVID-19 shutdown. *Science* **370**, 575–579 (2020).

9. Quesada-Rodríguez, C., Orientale, C., Diaz-Orozco, J. & Sellés-Ríos, B. Impact of 2020 COVID-19 lockdown on environmental education and leatherback sea turtle (Dermochelys coriacea) nesting monitoring in Pacuare Reserve, Costa Rica. *Biological Conservation* **255**, 108981 (2021).

10. Manenti, R. *et al.* The good, the bad and the ugly of COVID-19 lockdown effects on wildlife conservation: Insights from the first European locked down country. *Biological Conservation* **249**, 108728 (2020).

11. Koju, N. P., Kandel, R. C., Acharya, H. B., Dhakal, B. K. & Bhuju, D. R. COVID-19 lockdown frees wildlife to roam but increases poaching threats in Nepal. *Ecology and Evolution* **n/a**,.

12. Lu, M. *et al.* Does public fear that bats spread COVID-19 jeopardize bat conservation? *Biol Conserv* **254**, 108952 (2021).

13. Lopucki, R., Kitowski, I., Perlińska-Teresiak, M. & Klich, D. How Is Wildlife Affected by the COVID-19 Pandemic? Lockdown Effect on the Road Mortality of Hedgehogs. *Animals* **11**, 868 (2021).

14. Seress, G. *et al.* Contrasting effects of the COVID-19 lockdown on urban birds’ reproductive success in two cities. *Scientific reports* **11**, 1–10 (2021).

15. Cresswell, W. & Mccleery, R. How great tits maintain synchronization of their hatch date with food supply in response to long-term variability in temperature. *Journal of Animal Ecology* **72**, 356–366 (2003).
